# Effects of board characteristics on information asymmetry: Evidence from the alternative investment market^☆^^☆☆^

**DOI:** 10.1016/j.heliyon.2023.e16510

**Published:** 2023-05-29

**Authors:** Abdulateif A. Almulhim

**Affiliations:** Finance Department, Business School, King Faisal University, Al-ahsa, 31982, Saudi Arabia

**Keywords:** Corporate governance, Board characteristics, Information asymmetry, Information disclosure

## Abstract

This study aimed to investigate the effect of board characteristics on information asymmetry as well as examining whether the disclosure environment moderates the association between board structure and the information asymmetry of listed firms in the UK. We primarily focus on six characteristics of board composition (board size, board independence, board financial expertise, board busyness, CEO duality, and board gender diversity) and their impact on the bid–ask spread (employed as a proxy of information asymmetry). This study used the ordinary least squares (OLS) model to examine these associations. Moreover, we used system GMM and lag estimation models to test for endogeneity problems. Using a sample of 5950 observations representing the non-financial firms listed on the Alternative Investment Market (AIM) for 10 years from 2010 to 2019, we found a negative and significant relationship between board size; board independence; and female directors and information asymmetry. However, board busyness and CEO duality are positively related to information asymmetry. Furthermore, we demonstrate that information disclosure moderates the relationship between board characteristics and information asymmetry; that is, board size, independent directors, and female directors mitigate information asymmetry by improving the level of information disclosure. By contrast, busy directors and CEO duality increase the problem of information asymmetry by reducing firms’ information disclosure. The results of this study have implications for UK regulators, firm boards of directors, and firm stakeholders.

## 1 Introduction

In this study, we examine the impact of board characteristics on a corporation's information asymmetry and how this association is moderated by the corporation's information disclosure. Regulators, shareholders, and corporations mostly respond to issues related to information asymmetry by pushing and encouraging the board of directors to exhibit greater transparency. Such beliefs indicate that effective board composition can lead to the enhanced disclosure of information and can mitigate asymmetric information [1]. Supporting this point of view, agency theory argues that information asymmetry can be reduced by increasing the level of disclosure [2]. In line with this idea, several studies have empirically showed an adverse correlation between information disclosure and asymmetric information [3,4]. This effect can be improved by the effective monitoring and controlling of the practices of boards of directors [1,5,6]. For instance, the greater independence of directors on boards can lead to an enhancement in transparency and a reduction in asymmetric information [1].

Information asymmetry increases between managers and stockholders when ownership is separated from control. This may encourage managers to exploit the wealth of the firm instead of achieving stockholders' interests [7]. Managers may also exploit their power in the firm and disclose specific information when it is advantageous to them [8]. Previous studies have debated that corporate governance practices mitigate manager behaviour, protect stockholders’ rights, reduce asymmetric information, and align the interests of managers with those of stockholders [9,10].

Corporate governance has been a vital topic in the literature of finance since the chain of collapses and financial scandals worldwide [11] such as Marconi in the UK and Enron in the US [12]. After the global financial crisis of 2008, policymakers, practitioners, and academics believed that the insufficient performance of boards of directors was one reason for the crisis [[13], [14], [15]]. The boards were also blamed for concentrating on short-term objectives instead of long-term ones [15]. Thus, the importance of corporate governance effectiveness has been increasingly debated and there have been calls for more development and reforms of the mechanisms of corporate governance. Most corporate governance codes worldwide assert the significant role played by boards of directors in managing and controlling organisations. For instance, the UK Corporate Governance Code (2010) concentrates heavily on boards of directors when expressing corporate governance quality.

Boards of directors are responsible for implementing and crafting strategic decisions; ensuring better control and monitoring; meeting the company's objectives; maximizing the company's value; and aligning the interests of other stakeholders with those of managers [16,17]. Hence, the effectiveness of a board of directors can play a vital role in mitigating the issue of information asymmetry between different parties in firms, thereby increasing the level of disclosure and transparency in firms [9,16].

Due to the apparent success of the Alternative Investment Market (AIM), many countries have attempted to replicate the AIM market system. For example, the Tokyo Stock Exchange (TSE) created the Tokyo AIM in 2009. This market was established as a joint venture between the TSE and the London Stock Exchange (LSE). Although the Tokyo AIM uses the same system as the UK AIM, the former has not achieved the massive momentum and success of the UK AIM because the number of admissions to the Tokyo AIM has been low. The LSE withdrew from the Tokyo AIM in 2012.^1^ Another form of sub-market is Borsa Italiana's market, which was founded in 1997. Due to low demand on the market, it merged with the LSE and changed its name to AIM Italia in 2012. The characteristics of AIM Italia are similar to those of the UK AIM in that they both have lighter regulations and less stringent procedures.^2^ Nasdaq First North is another example of a junior market founded in 2006. Similar to the AIM, companies can obtain admission by having a Certified Adviser (called a Nominated Advisor in the UK AIM). The main job of the Adviser is to make sure that listed companies comply with the market regulations.^3^ The several attempts to replicate the UK AIM system worldwide are considered obvious evidence of its success since its launch in June 1995.

This paper aims to investigate the effect of board characteristics as a vital internal mechanism of corporate governance with respect to information asymmetry as well as how the disclosure environment moderates the association between board structure and information asymmetry in the Alternative Investment Market (AIM). As such, we focus on six characteristics of board composition: board size, board independence, board financial expertise, board busyness, CEO duality, and board gender diversity. Using a sample of 5950 observations representing the non-financial firms listed on the AIM for 10 years from 2010 to 2019, we report that board size, board independence, and female directors negatively influence information asymmetry, whereas board busyness and CEO duality positively affect information asymmetry. Moreover, we show that information disclosure moderates the relationship between board characteristics and information asymmetry. In other words, board size, independent directors, and female directors reduce information asymmetry through increasing the level of information disclosure, whereas busy directors and CEO duality increase information asymmetry by obstructing disclosure.

We contribute to the literature in several ways. First, to the best of our knowledge, this paper is the first to explore the relationship between board characteristics and information asymmetry as well as how information disclosure moderates the relationship between board characteristics and information asymmetry focusing on small and medium-sized firms. The AIM is a sub-market of the LSE. Based on the LSE, the AIM is the world's most successful growth market. It has a distinctive market system that is distinguished by light disclosure requirements and regulation regimes commensurate with the size of the firms listed in the market^4^ along with the non-compulsory adoption of corporate governance.^5^ AIM corporations commit to an unparalleled governance code called the Quoted Companies Alliance (QCA), which is designed to be appropriate for the nature, structure, needs, and size of small firms.^6^ In addition, the shareholder protection of the AIM is not the same as the Main Market of both the US and the UK as these two markets are distinguished by high shareholder protection [18,19], whereas the AIM has low shareholder protection [ [19]]. Prior studies, e.g., Porta et al. [20], have posited that corporate governance effectiveness adds value to corporations in weak investor protection environments. Thus, it is vital to examine how board composition influences the phenomenon of asymmetric information in such firms.

Third, we address the possibility of a moderation effect of information disclosure on board characteristics and information asymmetry. To the best of our knowledge, only one study has examined whether the disclosure environment moderates the association between board structure and information asymmetry [1]. Their study, however, focused only on the role of board independence and how it affects information asymmetry through the disclosure environment.

The remainder of this paper is organised as follows: Part 2 presents the development of hypothesis and literature review; Part 3 indicates the estimation models and data; and lastly, Part 4 explains the results of the study.

## 2 Literature review and hypothesis development

### 2.1 Corporate governance

Agency theory suggests that firm managers may use the resources of a firm to achieve personal gain. This can lead to a difference between managers' and stockholders' interests. Since managers have greater knowledge about a firm than do stockholders, information asymmetry may occur. Managers may therefore have the chance to expropriate the firm's wealth for themselves. However, the prior literature argues that corporate governance mechanisms are an influential solution that may decrease conflict between these parties by controlling managers' behaviour and aligning both managers' and stockholders' goals [7]. In this regard, effective corporate governance is a perfect method for guaranteeing that different parties in organisations are monitored and hence that they are working toward maximizing the wealth of shareholders [21]. Prior studies have also debated that effective internal corporate governance mechanisms increase the level of disclosure, lower asymmetric information, and prevent the expropriation of stockholders [16,22].

### 2.2 Review of board characteristics and information asymmetry studies

According to Jensen and Meckling [7], agency problems occur due to differences in the interests of managers and stockholders. Stockholders cannot handle agency problems because of information asymmetry concerning the actions and impacts of managers. Since stockholders cannot completely control managers' behaviours (such as shirking or consuming personal perquisites), agency problems may not be solved [23]. Mechanisms of corporate governance are perfect for stockholders' monitoring of agency issues by altering managers' behaviour [24,25]. According to the literature, managers' actions may be mitigated by reducing the impacts of information asymmetry, which can be achieved by employing mechanisms of board structure. For instance, independent directors can prevent collusion prerequisites and the shirking behaviour of managers through their reputation and experience in the market [26]. Another example of such a mechanism is the separation of the CEO role and that of the chairman, which is important for enhancing the quality of monitoring [27,28]. Gender diversity is also a mechanism recommended by policymakers and previous studies since it can enhance the ability to control managers’ actions and provide information to investors [29,30]. These mechanisms could raise the level of information disclosure and enhance transparency in corporations, therefore mitigating information asymmetry [31]. The following sections discuss the association between board characteristics and information asymmetry.

#### 2.2.1 Board size and information asymmetry

Board size is a vital attribute that influences the effectiveness of a board [27]. There are two streams of opinion regarding the optimal board size. The first stream argues that boards with more directors are better able to control and monitor managerial decisions due to their abundant resources. Larger boards are characterised by having the necessary skills to facilitate greater discussion about corporate matters, assign tasks, and form diverse effective committees [32,33]. In this regard, Flaherty et al. [34] and Cai et al. [35] found that larger boards mitigate asymmetric information as well as decreasing opacity [36]. The second stream argues that boards with more directors may possess some weaknesses that affect their performance and efficiency, such as social loafing and free riding [27,37]. Moreover, large boards suffer from increased coordination and communication problems as well as an increase in conflicts between the CEO and shareholders, reducing information disclosure and increasing information asymmetry. This ultimately gives firm managers a greater opportunity to raise their benefits [27,38]. For example, Yermack [38] showed that CEO threats of dismissal and compensation strategies are less effective in firms with a large board.

Eisenberg et al. [39] noted that board size impact across different classes of corporations influences the range of explanations for this impact. They argued that the elements that drive the selection of structure and board size in small corporations may differ from the elements affecting board size in large corporations. For instance, small corporations are mostly closely held; thus, the effect of agency problems between managers and stockholders on decisions influencing structure and board size are perhaps less widespread in such corporations. Supporting their argument, Eisenberg et al. [39] found an adverse association between board size and performance in small Finnish firms. Since the targeting sample of this study is the AIM (a market that consist of small and medium-sized corporations), we build our hypothesis as follows.

H1 Small board size reduces information asymmetry.

#### 2.2.2 Board independence and information asymmetry

According to agency theory, having outside directors is considered one of the most effective mechanisms of board structure. Such directors are able to monitor firm management; protect and work for stockholders' interests; and deliver information to stockholders [23,[40], [41], [42]]. In addition, a board with a higher percentage of independent members is better able to align the managers' interests with those of stockholders and thus improve the value of the corporation [7,23,43]. Outside directors are influential due to their reputation and experience in the market as well as their ability to share and provide information to the firm [26]. Furthermore, resource dependence theory considers board members as a vital resource since they add value to the corporation [44,45]. Independent non-executive directors are able to smooth access to resources that are important for a corporation's success [45]. They may bring resources to the corporation such as legitimacy, access to key constituents (social groups, buyers, suppliers, etc), information, and skills [44]. They can also assist in executing the strategy development of the firm [46].

The previous literature has highlighted the important role of independent directors on boards. For example, Pass [47] noted that corporations with outside members can increase the level of firm accountability and integrity due to such directors caring about their reputation in the market. They can also evaluate opportunities and risks better than the firm's executives since they are busy carrying out and concentrating on day-to-day business. In addition, independent directors can discipline and control overbearing managers since they are not under the control of the managers [47].

Welker [3] argued that a higher number of outside directors on the board is associated with a reduction in asymmetric information since these directors are able to increase the level of transparency of the firm. Moreover, Goh et al. [1] found that board independence mitigates information asymmetry in the NASDAQ, AMEX, and NYSE stock exchanges. Similarly, Almulhim [48] indicated that board independence is negatively related to asymmetric information. Based on the above arguments, we build our hypothesis as follows.

H2 Board independence reduces information asymmetry.

#### 2.2.3 Board financial expertise and information asymmetry

The personal attributes of directors, such as working experience, are vital determinants of board effectiveness [49]. Professional expertise is crucial for members in order to fulfil their role of controlling the quality of financial disclosure [33,[50], [51], [52], [53]]. In this regard, Kroll et al. [49] noted that the vigilance of directors is not sufficient to monitor firm management; relevant working experience and knowledge are also important as they assist directors with their monitoring duties. In this regard, regulators and scholars have emphasised the importance of having directors with financial expertise on boards due to the large number of accounting scandals that corporations have been exposed to recently [54]. The lack of members with financial expertise on boards played a crucial role in the global financial crisis [55,56]. Prior studies showed that boards with financial expertise members can positively affect the behaviour of corporations regarding hedging policies [57]. These directors also reduce the time firms remain below the base capital ratio; thus, directors with financial expertise assist in managing firm capital and improve stability [58].

In addition, directors with financial expertise are considered better able to guide managers to avoid risks than are other directors [59] and facilitate the acquisition of information regarding financial transactions at a low cost [60]. The existence of financial expertise on boards also reduces the investigation costs of financial information and therefore facilities access to external financing [61,62]. Empirically, Hau and Thum [63] found that German corporations with more financial expertise on boards achieved better performance during the financial crisis. Mahdy et al. [64] found that directors with financial expertise adversely affect information asymmetry. Based on the above arguments, we build our hypothesis as follows.

H3 Board financial expertise reduces information asymmetry.

#### 2.2.4 Board busyness and information asymmetry

The literature on board characteristics presents two opinions about busy members on boards (reputational impact and busyness impact) [65]. With regard to reputational impact, busy directors with directorships on different boards improve decisions made by the board due to their experience and business connections in addition to their advisory role and controlling functions [66,67]. Busy members may improve acquisition performance [68]. Ferris et al. [69] showed that appointing a new busy director is associated with positive announcement returns.

On the other hand, busy directors may be considered to be too busy to put all their effort into the business or to fully concentrate on the business. This may lead to non-perfect corporate decisions and agency conflicts [70]. They may be overcommitted since they hold multiple memberships on several boards, hampering their ability to provide considerable managerial control [71,72]. Busy members are also related to ineffective corporate governance and thus decrease firm value [73,74]. Fich and Shivdasani [74] indicated that corporations that have busy members on their boards have lower CEO turnover during their poor performance. Moreover, they found an adverse correlation between busy members and market-to-book ratios. Such members are less likely to effectively advise and control managers, which negatively influences corporation performance [74]. Moreover, busy members are correlated with attendance issues at board meetings and immoderate CEO pay [72]. Furthermore, Almulhim [48] showed that busy directors have a positive and significant association with asymmetric information.

In accordance with this point of view, several regulators worldwide have recently imposed restrictions on the number of busy directors. According to a survey conducted by the Spencer and Stuart U.S. Board Index in 2012, approximately 74% of Standard and Poor's 500 corporations have limited the number of multiple memberships held by their board directors [70]. In addition, Institutional Shareholder Services (ISS) also recommend corporations limit the number of members who have multiple membership on their boards. Based on the arguments above, we build our hypothesis as follows.

H4 Board busyness increases information asymmetry.

#### 2.2.5 CEO duality and information asymmetry

The chairman and the CEO are considered to be the crucial leaders of corporations. The chairman is responsible for addressing strategic matters, setting agendas, leading the meetings of the board, representing the firm in front of other outside corporations, etc., whereas the CEO is responsible for managing the corporation and its daily operational responsibilities [75]. Normally, the posts of the chairman and CEO are held by two different individuals; however, it is not uncommon to find corporations where one individual holds the dual role of chairman and CEO of the board (referred to as duality). Agency theory recommends that boards of corporations should be independent to avoid managerial violation [23,43]. Since CEO duality is not consistent with this recommendation, agency theory argues that CEO duality adversely influences the performance of corporations [27,76].

Based on this, previous studies have debated that separating the roles of CEO and chairman is important for enhancing monitoring quality [27,28]. By contrast, CEO duality may weaken the effectiveness of the board in monitoring and controlling functions [77,78], thereby increasing agency costs and information asymmetry [79]; that is, duality may mitigate the board's ability to accomplish its governance and oversight roles and to spread corporate information to stakeholders [80]. Corporations with the same chairman and CEO may have a lower level of voluntary disclosure [81], which may result in high information asymmetry and a lack of transparency [82,83]. Empirically, Hsu et al. [84] indicated that CEO duality negatively impacts the performance of firms when information asymmetry is high. Based on the above arguments, we build our hypothesis as follows.

H5 CEO duality increases information asymmetry.

#### 2.2.6 Gender diversity and information asymmetry

The representation of female directors on boards has been low in the past, and in response various regulators have recommended corporations appoint female directors on their boards. For instance, the UK Corporate Governance Code states that boards should promote the diversity of gender; ethnic and social backgrounds; and personal and cognitive strengths. Such recommendations have led to an increase in the amount of research regarding the vital role of women directors on boards. Several theories support the concept of diversity in corporations. For example, human capital theory investigates the impact of a person's cumulative repository of experience, education, and skills on their cognitive capabilities and developing productivity, which lead to benefits for both the person and the corporation [85]. In the same vein, diversity on boards is seen as an important resource for corporations. In addition, resource dependence theory argues that board linkages provide communication channels and legitimacy; that is, the diversity of members provides various benefits for the corporation [86]. Agency theory also explores the roles of asymmetric information and different objectives in the association between managers and stockholders, suggesting the former might not work to achieve stockholders' interests [7,43]. This theory argues that gender diversity can mitigate agency conflict [87].

Prior studies in the literature show that board diversity raises both the quality and quantity of information disclosure. For instance, Adams and Ferreira [88] reported that women on a board of directors are more likely to be chosen for committees such as corporate governance and audit committees. This leads them to become directly involved in increasing the level of transparency. Furthermore, women who sit on boards have been reported to encourage more effectual communications with shareholders [89], which increases the quality and diffusion of information about a firm [29,30] and reduces asymmetric information [4,90]. Empirical evidence has also shown that female directors reduce information asymmetry between inside and outside shareholders [91,87]. We use female directors as a proxy for the diversity of the board. Based on the above arguments, the following hypothesis is developed.

H6 Gender diversity reduces information asymmetry.

### 2.3 Moderating impact of information disclosure on board characteristics and information asymmetry associations

In addition to exploring the effects of board characteristics on information asymmetry, we argue that board characteristics could affect asymmetric information through their impact on the disclosure of firms. Agency theory suggests that increased transparency and better disclosure are likely to mitigate asymmetric information [2]. In line with this argument, several studies have shown empirically that effective monitoring and controlling of the practices of boards of directors is related to higher levels of disclosure and lower levels of asymmetric [1,3,4,6,92,93].

Thus, we examine whether information disclosure moderates the association between board characteristics and asymmetric information. One topic that seems to have not yet been covered by the literature is the comprehensive analysis of the characteristics of boards of directors (we employ six board characteristics—board size, board independence, board financial expertise, board busyness, CEO duality, and board diversity—rather than concentrating on certain board mechanisms). In this study, we explore whether information disclosure moderates the impact of board structure on information asymmetry. By conducting such an investigation, we can gain a better understanding of the roles of these comprehensive mechanisms of board structure. Based on the above arguments, the following hypothesis is developed.

H7 Information disclosure moderates the impact of board characteristics on information asymmetry.

## 3 Data and methodology

### 3.1 Data sample

This study was conducted on the UK Stock Exchange, particularly the AIM, and the sample comprised the non-financial companies^7^ of the FTSE AIM All-Share Index over a 10-year period between 2010 and 2019. We covered this period to avert the possible effects of unobservable external macroeconomic impacts during the financial crisis of 2008–2009 and the COVID-19 pandemic. The initial sample included 906 corporations. However, this paper excluded financial corporations since their regulatory operating and environments are different from those of non-financial corporations. Hence, corporations from the financial industry were excluded (284 corporations). This reduced the sample to 660 corporations. Moreover, corporations with missing information asymmetry or that were missing board data for at least 3 consecutive years were dropped from the sample (27 corporations) [94]. The final sample with an unbalanced panel of 595 non-financial corporations (e.g., 5950 observations over time) is presented in Table 1.

**Table 1:** Industry classification of sample corporations.

Industries	Number of Firms	Proportion of Firms Per Industry
Industrials	117	19.66
Basic Materials	113	18.99
Technology	93	15.63
Oil & Gas	82	13.78
Consumer Services	72	12.10
Health Care	61	10.25
Consumer Goods	40	6.72
Telecommunication	9	1.52
Utilities	8	1.35
**Total**	595	100

For the dependent variable, information asymmetry (proxied by the bid–ask spread) was obtained from the Datastream database. Financial data were collected from the Datastream database. Finally, data on board characteristics were obtained from the BoardEx database, whereas data on information disclosure (proxied by analyst following) were collected from the Bloomberg database.

### 3.2 Measures

#### 3.2.1 Information asymmetry variable

We followed prior studies in the literature [1,3,93,95] and used the bid–ask spread as a proxy for information asymmetry. Previous studies, such as Glosten and Harris [96], Kyle [97], and Copeland and Galai [98] have argued that market makers widen the bid–ask spread when informed investors trade. We used the following equation to measure the bid–ask spread (QBAS).

(1) $$MIDP=\frac{\left(ASK+BID\right)}{2}$$

(2) $$QBAS=\frac{\left(ASK\;–\;BID\right)}{MIDP}$$

As seen in Eq. (1). Eq. (2) that MIDP is the midpoint of quote, QBAS is the quoted bid–ask spread, ASK is the asking price, and BID is the bid price.

#### 3.2.2 Information disclosure variable

Analyst following (AF) has been used by previous studies as a representative of information disclosure [1,64,99,100]. A corporation's analyst following is employed as a representative of the level of non‐governance disclosures and it reflects the communication of a corporation with its analyst following [ [101]].

#### 3.2.3 Board characteristics variables

This paper studies six mechanisms of board composition (board size, board independence, board financial expertise, board busyness, CEO duality, and board gender diversity) in order to explore their impact on information asymmetry. Board size (BoDS) is defined as the aggregate number of members on the board [102]; board independence (BINED) is defined as the total number of independent directors to the total number of directors on the board [[103], [104]] ; board financial expertise (BFE) is defined as the total number of directors with accounting and finance degrees to the total number of directors on the board [62,105]; board busyness (BBusy) is described as the number of directors that serve on two or above boards to the total aggregate number of board directors [106]; CEO duality (CEOD) is measured by a dummy variable, which takes 1 if the CEO and the chairman are the same person and 0 otherwise [75]; and board gender diversity (BDG) is measured as the number of female directors to the total aggregate number of board directors [91,87].

#### 3.2.4 Control variables

Several variables for the financial characteristics of corporations were employed to investigate the association between board characteristics and information asymmetry and disclosure: corporation size, corporation age, financial leverage, expenditure on research and development, and asset tangibility. Corporation size (FSIZE) is the total assets of a corporation. Generally, small corporations tend to have higher information asymmetry since they are more likely to disclose less information to the public [107]. Corporation age (FAGE) is the number of years since a corporation was first listed on the AIM market. Generally, younger corporations have a smaller investor base, which could lead to increased asymmetric information [108]. Financial leverage (FLV) is described as the percentage of total liabilities to total assets. Frequently, corporations with more asymmetric information generally have higher costs for equity issuance. This may lead corporations to prefer debt financing instead of equity financing. Thus, corporations with high levels of asymmetric information have higher financial leverage [109]. Expenditure on Research and development (R&D) is measured as the expenditure on development and research to sales. Unlike asset tangibility, payoffs from development and research expenditure are hard to observe. This can worsen asymmetric information problems [9]. Asset tangibility (FAT) is described as the ratio of net property, plant, and equipment to total assets. Prior studies posit that tangible assets mitigate the problems of asymmetric information as their payoffs are easier to observe [9]. Finally, we controlled for the exclusive effects of time factors and industry-level factors by employing dummy variables for year and industry studied [110]. Table 2 provides the definitions and calculations of the variables.

**Table 2:** Variable definitions and calculations of information asymmetry, disclosure, board characteristics, and financial variables.

Variable	Definition	Data Source
Dependent variables		
Quoted bid–ask spread (QBAS)	QBAS = ask price ₋ bid price/midpoint price.	Own calculation
**Independent variables**		
Analyst following (AF)	AF is the number of analysts who follow or observe an AIM corporation.	BoardEx
Board size (BoDS)	BoDS is the aggregate number of members on the board.	BoardEx
Board independence (BINED)	BINED is the total number of independent directors to the total number of directors on the board.	BoardEx
Board financial expertise (BFE)	BFE is the total number of directors with accounting and finance degrees to the total number of directors on the board.	BoardEx
Board busyness (BBusy)	BBusy is the number of directors that serve on two or above boards to the total aggregate number of board directors.	BoardEx
CEO duality (CEOD)	CEOD takes 1 if the CEO and chairman are the same person and 0 otherwise.	BoardEx
Board gender diversity (BDG)	BDG is the number of female directors to the total aggregate number of board directors.	BoardEx
**Financial variables**		
Corporation size (FSIZE)	FSIZE is the total assets of the AIM corporation.	Datastream
Corporation age (FAGE)	FAGE is the number of years since the corporation was listed on the market.	AIM firms' websites
Financial leverage (FLV)	FLV is the proportion of total liabilities to total assets.	Datastream
Expenditure on research and development (R&D)	R&D is the expenditure on development and research to sales.	Datastream
Asset tangibility (FAT)	FAT is the ratio of net property, plant, and equipment to total assets.	Datastream

### 3.3 Estimation models

The primary estimation model used in this study was the pooled OLS regression using different board structure variables in addition to the financial variables and industry and year dummies. To control for the problem of heteroscedasticity, we employed robust standard errors in our models. The lag and system GMM estimations were also used as a robustness check to control for endogeneity problems. To empirically explore the relationship between board characteristics and information asymmetry, we used two different models as follows:

First, measuring the association between board characteristics and asymmetric information is provided in Eq. (3).

(3) QBASit = β0 + β1 BoDSit + β2 BINEDit + β3 BFEit + β4 BBusyit + β5 CEODit + β6 BDGit + β7 FSIZEit + β8 FAGEit + β9 FLVit + β10 R&Dit + β11 FATit + εit

where *QBASit* represents the bid–ask spread; *BoDSit* represents board size; *BINEDit* represents board independence; *BFEit* represents board financial expertise; *BBusyit* represents board busyness; *CEODit* represents CEO duality; and *BDGit* represents board gender diversity. The control variables include *FSIZEit*, *FAGEit*, *FLVit*, *R&Dit*, and *FATit* (corporation size; corporation age; financial leverage; expenditure on research and development; and asset tangibility, respectively), and *εit* represents the error terms.

(4) QBASit = β0 + β1 BAFit + β2 BoDSit + β3 BINEDit + β4 BFEit + β5 BBusyit + β6 CEODit + β7 BDGit + β8 (BoDS*AF)it + β9 (BINED*AF)it + β10 (BFE*AF)it + β11 (BBUSY*AF)it + β12 (CEOD*AF)it + β13 (BDG*AF)it +β14 FSIZEit + β15 FAGEit + β16 FLVit + β17 R&Dit + β18 FATit + εit

where (BoDS*AF)it represents the interaction terms between board size and information disclosure; (BINED*AF)it represents the interaction terms between board independence and information disclosure; (BFE*AF)it represents the interaction terms between board financial expertise and information disclosure; (BBUSY*AF)it represents the interaction terms between board busyness and information disclosure; (CEOD*AF)it represents the interaction terms between CEO duality and information disclosure; (BDG*AF)it represents the interaction terms between board gender diversity and information disclosure; and the rest of variables are defined in formula (3).

## 4 Results

### 4.1 Descriptive statistics and correlations

Table 3 shows the descriptive statistics of the information asymmetry and disclosure measures, board characteristics, and controlling variables. For the information asymmetry variable, the average value and standard deviation of the bid–ask spread (QBAS) were 7.558% and 6.744%, respectively. The average value and standard deviation of the information disclosure variable (AF) were 5.027 and 4.923 respectively. With regard to the explanatory variables, BoDS had an average and a standard deviation of 5.4 and 1.51 respectively, while the minimum and the maximum values were 3 and 14 respectively. Also, the average percentage of BINED was 51.43%; the minimum percentage was 0%; and the maximum percentage was 100%. In addition, BFE had a standard deviation of 15.91% with an average of 39.65%; and the maximum value was 100%. Moreover, the average percentage of BBusy was 28.22% with a minimum percentage of 0%; and a maximum percentage of 100%. The average value of CEOD was 0.060, whereas the proportion of BDG was 5.50% with a minimum value of 0% and a maximum value of 50%. Regarding the financial variables, the average FSIZE was 33.39 million with a standard deviation of 117. The average FAGE was 11.5 years, with the oldest corporation being listed 24 years ago. The maximum-minimum value of AIM firm FLV was 121%–0% with an average of 59%. The average of R&D was 6.8% and its standard deviation was 18%. Furthermore, AIM firms had a FATR average of 17.19% with a maximum-minimum percentage of 99%–0%, respectively.

**Table 3:** Descriptive statistics.

Variables	Observation	Mean	SD	Min	Max
QBAS	5935	7.558	6.644	0.074	63.476
AF	5935	5.027	4.923	0.000	22.00
BoDS	5935	5.455	1.513	3.000	14.00
BINED	5935	51.43	19.06	0.000	100.0
BFE	5935	39.65	15.91	0.000	100.0
BBusy	5935	28.22	16.71	0.000	100.0
CEOD	5,9 35	0.060	0.227	0.000	1.000
BDG	5935	5.506	9.093	0.000	50.00
FSIZE	5935	33.39	117.2	9.520	72.40
FAGE	5935	11.55	5.116	1.000	24.00
FLV	5935	59.88	317.3	0.000	121.4
R&D	5935	6.835	18.45	0.000	69.87
FATR	5935	17.19	23.37	0.000	99.71

Note: BAS is bid–ask spread, AF is analyst following, BoDS is board size, BINED is board independence, BFE is board financial expertise, BBusy is board busyness, CEOD is CEO duality, BDG is board diversity gender, FSIZE is firm size, FAGE is firm age, FLV is financial leverage, R&D is Research and development expenditure, and FATR is assets tangibility.

To check whether our study variables had any multicollinearity problems, we employed two measurements: the variance inflation factors (VIF) and Pearson matrix (Table 4, Table 5, respectively). According to the correlation matrix, the highest correlation among the explanatory variables was between BFE and BBusy (70%); that is, directors with financial expertise were more likely to serve on many boards. In addition, the correlation between BINED and BBusy was the second highest association at 45%. Thus, independent directors were also more likely to serve on many boards. Based on the VIFs presented in Table 5, our model had no multicollinearity problems since all the values were less than 10 [111].

**Table 4:** Pearson correlation matrix for the relationship between board characteristics, information asymmetry and disclosure.

	QBAS	AF	BoDS	BINED	BFE	BBusy	CEOD	BDG	FSIZE	FAGE	FLV	R&D	FATR
QBAS	1.0000												
AF	−0.3682*	1.0000											
BoDS	−0.3378*	0.2768*	1.0000										
BINED	−0.1838*	0.2210*	0.2312*	1.0000									
BFE	−0.0588*	0.1051*	0.1327*	0.3086*	1.0000								
BBusy	−0.0736*	0.0972*	0.2233*	0.4556*	0.7069*	1.0000							
CEOD	0.0613*	−0.0328*	0.0159	0.0614*	0.0638*	0.0739*	1.0000						
BDG	−0.0330*	0.0620*	0.1472*	−0.0131	−0.0529*	−0.0385*	−0.0498*	1.0000					
FSIZE	−0.2591*	0.3637*	0.2297*	0.1608*	0.0553*	0.0622*	−0.0348*	0.1423*	1.0000				
FAGE	0.0547*	0.0031	−0.0178	−0.0157	−0.0761*	−0.0726*	−0.0536*	0.0432*	0.0203	1.0000			
FLV	0.0651*	0.0079	−0.0430*	0.0186	0.0276*	0.0011	0.0139	0.0074	−0.0131	0.0106	1.0000		
R&D	−0.0171	0.0040	0.0418*	−0.0007	0.0282*	0.0383*	−0.0432*	−0.0331*	−0.0091	0.0078	−0.0124	1.0000	
FATR	−0.1189*	0.1489*	0.0923*	0.1395*	0.1185*	0.1226*	0.0368*	−0.0336*	0.0344*	−0.0658*	−0.0208	−0.0550*	1.0000

Note: BAS is bid–ask spread, AF is analyst following, BoDS is board size, BINED is board independence, BFE is board financial expertise, BBusy is board busyness, CEOD is CEO duality, BDG is board diversity gender, FSIZE is firm size, FAGE is firm age, FLV is financial leverage, R&D is Research and development expenditure, and FATR is assets tangibility.

Note: *means significance level at 5%.

**Table 5:** Variance inflation factors (VIF).

Variance Inflation Factors (VIF)	
Variable	QBAS
BBusy	2.38
BFE	2.02
FSIZE	1.74
BINED	1.42
BoDS	1.41
FATR	1.39
AF	1.24
FLV	1.14
CEOD	1.04
BDG	1.04
FAGE	1.03
R&D	1.02
**Mean VIF**	***1.40***

Note: BAS is bid–ask spread, AF is analyst following, BoDS is board size, BINED is board independence, BFE is board financial expertise, BBusy is board busyness, CEOD is CEO duality, BDG is board diversity gender, FSIZE is firm size, FAGE is firm age, FLV is financial leverage, R&D is Research and development expenditure, and FATR is assets tangibility.

### 4.2 Multivariate analyses

#### 4.2.1 Board characteristics and information asymmetry

Table 6 shows the regression findings for the association between board characteristics and information asymmetry. BoDS was negatively and significantly related to QBAS at the 1% significance level. This means that the larger the size of the board, the lower the information asymmetry. This is in line with the results of [34,35] and is also consistent with the view that larger boards mostly have more resources to control skills and managerial performance so as to form many effectual committees, assign tasks, and allow for greater argument on corporate issues. This may lead to lower information asymmetry and greater transparency [32,33].

**Table 6:** Effects of board characteristics on information asymmetry.

VARIABLES	QBAS
	
BoDS	−0.345***
	(0.065)
BINED	−0.021***
	(0.004)
BFE	0.045
	(0.067)
BBusy	0.011*
	(0.006)
CEOD	0.050***
	(0.008)
BDG	−0.139*
	(0.078)
FSIZE	−0.385***
	(0.011)
FAGE	−0.012
	(0.011)
FLV	0.027***
	(0.005)
R&D	0.0126**
	(0.005)
FATR	−0.010***
	(0.002)
Constant	−1.580***
	(0.0512)
	
Observations	5905
R-squared	0.615
YEARS FE	*YES*
INDUSTRY FE	*YES*

Note: ***, **, and * denote significance levels of 1%, 5%, and 10%, respectively.

Robust standard errors are found in parentheses.

BAS is bid–ask spread, BoDS is board size, BINED is board independence, BFE is board financial expertise, BBusy is board busyness, CEOD is CEO duality, BDG is board diversity gender, FSIZE is firm size, FAGE is firm age, FLV is financial leverage, R&D is Research and development expenditure, and FATR is assets tangibility.

Board independence (BINED) also showed a significant and negative correlation with QBAS at the 1% significance level. Goh et al. [1] and Almulhim [48] also obtained the same result. This negative relationship is in line with the idea that a higher proportion of independent members on the board can help to align managers' and shareholders’ interests and hence improve the value of the corporation [7,23,43]. Independent members are seen as more influential due to their reputation, experience, and ability to provide information and ideas for the firm [26].

Moreover, it was found that BFE had no association with information asymmetry. BBusy was positively and significantly related to QBAS at the 10% significance level. BBusy therefore had a positive relationship with information asymmetry. This positive result supports the busyness hypothesis argument, which states that directors with multiple seats on boards do not have enough time for each firm, leading to an increase in agency problems and imperfect corporate decisions. These directors are overcommitted, which may inhibit their ability to pay attention and provide adequate managerial controlling. This may weaken a firm's performance [[70], [71], [72]].

Furthermore, CEOD had a positive and significant correlation with QBAS at the 1% significance level. This finding is consistent with the findings of Finkelstein and D'aveni [77] and Tuggle et al. [78], which suggested that CEOD may weaken the effectiveness of the board in monitoring and controlling functions and hence increase agency costs and information asymmetry [79]. In addition, the existence of female members on the board (BDG) was negatively and significantly related to QBAS at the 10% significance level. Female directors therefore have an adverse relationship with asymmetric information. This negative result is consistent with the findings of Abad et al. [91] and Loukil et al. [87], which demonstrated that female directors reduce information asymmetry between inside and outside shareholders.

With respect to the controlling variables, it is observed that FSIZE was negatively associated with QBAS at the 1% significance level. By contrast, FAGE had no relationship with information asymmetry. FLV had a positive association with QBAS at the 1% significance level. Thus, firms with high leverage exhibited information asymmetry. Although R&D had a positive association with QBAS at the 5% significance level, FATR was negatively related to QBAS at the 1% significance level. These results are in line with the argument of Chung et al. [9] that payoffs from R&D expenditure are difficult to observe. Thus, they can worsen asymmetric information problems. By contrast, asset tangibility mitigates the problems of asymmetric information as its payoffs are easier to observe.

#### 4.2.2 How information disclosure moderates the impact of board characteristics on information asymmetry

The data in Table 6 prove that different board practices have different influences on information asymmetry. An extensively accepted debate in the literature is that effective monitoring and control through the influential practices of boards of directors can mitigate information asymmetry through higher information disclosure levels [1,5,6]. Thus, the following section aims to explore whether board characteristics affect information asymmetry through information disclosure. Table 7 indicates that board size, independent directors, and female directors reduce information asymmetry through increasing the level of information disclosure, whereas busy directors and CEO duality increase information asymmetry by obstructing disclosure.

**Table 7:** Moderating effects of information disclosure on board characteristics and information asymmetry.

VARIABLES	QBAS
AF	−0.108***
	(0.012)
BoDS	−0.105***
	(0.009)
BINED	−0.031***
	(0.010)
BFE	−0.293***
	(0.068)
BBusy	0.033***
	(0.009)
CEOD	0.126***
	(0.016)
BDG	0.009***
	(0.002)
BoDS*AF	0.002*
	(0.001)
BIND*AF	0.008***
	(0.001)
BFE*AF	0.003
	(0.011)
Bbusy*AF	−0.002*
	(0.001)
CEOD*AF	−0.006**
	(0.002)
BDG*AF	−0.116***
	(0.021)
FSIZE	−0.135***
	(0.011)
FAGE	0.083***
	(0.0137)
FLV	0.059***
	(0.007)
R&D	−0.087
	(0.061)
FATR	−0.014***
	(0.002)
Constant	−0.714***
	(0.108)
	
Observations	5905
R-squared	0.497
YEARS FE	*YES*
INDUSTRY FE	*YES*

Note: ***, **, and * denote significance levels of 1%, 5%, and 10%, respectively.

Robust standard errors are found in parentheses.

BAS is bid–ask spread, AF is analyst following, BoDS is board size, BINED is board independence, BFE is board financial expertise, BBusy is board busyness, CEOD is CEO duality, BDG is board diversity gender, FSIZE is firm size, FAGE is firm age, FLV is financial leverage, R&D is Research and development expenditure, and FATR is assets tangibility.

#### 4.2.3 Board characteristics and information asymmetry (robustness check)

Table 8 re-examines the link between board characteristics and information asymmetry as well as the moderation effects of information disclosure on board characteristics and information asymmetry after controlling for the problems of endogeneity, employing both the lag estimation model and system GMM model. Panels A and B represent the lag estimation model and system GMM model for the association between board characteristics and information asymmetry, respectively, whereas panels C and D represent the lag estimation model and system GMM model for the moderation effects of information disclosure on board characteristics and information asymmetry, respectively. The results presented are the same as those of the main estimation models in Table 6, Table 7

**Table 8:** Effects of board characteristics on information asymmetry (robustness check).

	LAG	GMM	LAG	GMM
VARIABLES	(A) QBAS	(B) QBAS	(C) QBAS	(D) QBAS
AF			−0.118***	−0.045***
			(0.013)	(0.017)
BoDS	−0.346***	−0.079**	−0.103***	−0.023*
	(0.064)	(0.031)	(0.009)	(0.013)
BINED	−0.021***	−0.099*	−0.037***	−0.067***
	(0.004)	(0.050)	(0.010)	(0.018)
BFE	0.018	−0.014	−0.333***	0.004
	(0.066)	(0.021)	(0.070)	(0.011)
BBusy	0.016***	0.013**	0.039***	0.058***
	(0.006)	(0.005)	(0.009)	(0.018)
CEOD	0.062***	0.084*	0.141***	−0.273***
	(0.008)	(0.050)	(0.016)	(0.026)
BDG	−0.149*	−1.465*	0.370***	−0.699**
	(0.079)	(0.833)	(0.014)	(0.275)
BoDS*AF			0.003**	−0.008***
			(0.001)	(0.002)
BIND*AF			0.010***	−0.005***
			(0.002)	(0.002)
BFE*AF			0.003	0.001
			(0.012)	(0.002)
Bbusy*AF			−0.003**	0.008***
			(0.001)	(0.002)
CEOD*AF			−0.006**	0.084***
			(0.002)	(0.075)
BDG*AF			−0.106***	0.083***
			(0.020)	(0.032)
FSIZE	−0.383***	−0.364***	−0.134***	−0.344***
	(0.012)	(0.032)	(0.012)	(0.011)
FAGE	0.084	−0.136***	0.107***	−0.144***
	(0.126)	(0.048)	(0.014)	(0.021)
FLV	0.022***	0.069**	0.044***	0.034***
	(0.005)	(0.027)	(0.007)	(0.005)
R&D	0.016***	0.007	−0.028	0.085*
	(0.005)	(0.283)	(0.060)	(0.050)
FATR	−0.096***	−0.017	−0.011***	−0.079
	(0.026)	(0.011)	(0.002)	(0.048)
Constant	−1.649***	−1.307***	−0.916***	−1.351***
	(0.054)	(0.292)	(0.114)	(0.124)
				
Observations	5313	5905	5313	5905
R-squared	0.625		0.512	
YEARS FE	*YES*	*YES*	*YES*	*YES*
INDUSTRY FE	*YES*	*YES*	*YES*	*YES*
AR (1) test (p-value)		0.000		0.000
AR (2) test (p-value)		0.489		0.286
Hansen test of over-identification (p-value)		0.843		0.846

Note: ***, **, and * denote significance levels of 1%, 5%, and 10%, respectively.

Robust standard errors are found in parentheses.

QBAS is bid-ask spread, AF is analyst following, BoDS is board size, BINED is board independence, BFE is board financial expertise, BBusy is board busyness, CEOD is CEO duality, BDG is board diversity gender, FSIZE is firm size, FAGE is firm age, FLV is financial leverage, R&D is Research and development expenditure, and FATR is assets tangibility.

## 5 Conclusions

The target of this study was to examine the link between board characteristics and information asymmetry as well as the moderating impact of information disclosure on board characteristics and information asymmetry. The studied sample contained non-financial firms listed on the AIM for ten years from 2010 to 2019. This topic had not previously been investigated with respect to the AIM, a market that is characterized by small and medium-sized firms. The AIM is the world's most successful growth market. The market's distinctive system, light disclosure, regulation, and corporate governance are designed to be appropriate for the nature, structure, needs, and size of small firms. In addition, our contribution was extended by analysing the comprehensive characteristics of boards of directors (board size, board independence, board financial expertise, board busyness, CEO duality, and board diversity) rather than concentrating on particular board mechanisms. Regarding methodology, we used OLS as the estimation model of this study together with lag and system GMM estimations in order to address any possible endogeneity issues.

With respect to the results of this study, we found a negative and significant relationship between board size; board independence; and female directors and information asymmetry. However, board busyness and CEO duality were positively related to information asymmetry. Furthermore, we demonstrated that information disclosure moderates the relationship between board characteristics and information asymmetry; specifically, board size, independent directors, and female directors mitigate information asymmetry by improving the level of information disclosure. By contrast, busy directors and CEO duality increase the problem of information asymmetry by reducing firm information disclosure. These results were additionally confirmed by both lag and system GMM estimations.

Overall, this paper has implications for corporations, regulators, and policymakers. The results reported in this study can provide guidance and direction to the regulators and top management of corporations with respect to the effective composition of a board of directors. That is, having sufficient representation of independent members, female members, and separating the roles of chairman and CEO could enhance their controlling role in mitigation asymmetric information, increasing the level of information disclosure, and reducing possible conflicts of interest between managers and stockholders. In addition, our results may draw the attention of policymakers and regulators in second tier market like the AIM to limit appointing busy directors by determining a maximum number of busy directors on board.

One of the limitations of this study is that the measures of information asymmetry and information disclosure are proxied by one indicator. Future research can employ more proxies for information asymmetry and information disclosure to examine their effects on board structure. In addition, we defined board busyness as the number of directors that serve on two or above boards to the total aggregate number of board directors. We found a positive association between busy directors and asymmetric information. Researchers can further classify busy directors based on the number of seats on boards they possess (e.g., three, four, etc). This may provide a different perception of the link between board busyness and asymmetric information. Furthermore, future studies may investigate the relationship between board characteristics and information asymmetry during financial crises (e.g., COVID-19 pandemic).

## Author contribution statement

Abdulateif A Almulhim: Conceived and designed the experiments; Performed the experiments; Analyzed and interpreted the data; Contributed reagents, materials, analysis tools or data; Wrote the paper.

## Data availability statement

Data will be made available on request.

## Declaration of competing interest

The authors declare that they have no known competing financial interests or personal relationships that could have appeared to influence the work reported in this paper.

## Footnotes

1 Tokyo Stock Exchange (TSE) website: http://www.jpx.co.jp/english/equities/products/tpm/outline/index.html.

2 Borsa Italiana website: https://www.borsaitaliana.it/borsaitaliana/storia/storia/consolidamento.en.htm.

3 Nasdaq First North website: https://www.nasdaq.com/solutions/nasdaq-first-north-growth-market.

4 AIM firms have *“no minimum market capitalization, no trading record requirement, no prescribed level of shares to be in public hands, no prior shareholder approval for most transactions, admission documents not pre-vetted by the Exchange nor by the UKLA in most circumstances”* (LSE, 2010a, p. 6).

5 LSE (2016, p. 12) states that *“details of the corporate governance code that the AIM company has decided to apply, how the AIM company complies with that code, or if no code has been adopted this should be stated together with its current corporate governance arrangements”*.

6 *“The QCA's Corporate Governance Guidelines for AIM Companies have become a widely recognised benchmark for SME corporate governance. We fully support the use of these Guidelines to achieve a level of corporate governance measures appropriate for an AIM company”* (LSE, 2010b, p. 1).

7 In 2019, non-financial companies were able to raise new money about 388 (£m) compared with financial companies that raised only 39 (£m). In addition, the total number of companies that listed on the AIM in 2019 are 21 new firms, where 81% of them are non-financial (LSE, AIM, reports) link: https://www.londonstockexchange.com/reports?tab=aim.
